# Variation of spatiotemporal parameters in school children carrying different backpack loads: a cross sectional study

**DOI:** 10.1038/s41598-019-48675-3

**Published:** 2019-08-21

**Authors:** Joaquin Paez-Moguer, Jesus Montes-Alguacil, Irene Garcia-Paya, Miguel Medina-Alcantara, Angela Margaret Evans, Gabriel Gijon-Nogueron

**Affiliations:** 1 Department of Nursing and Podiatry, University of Malaga, Malaga, Spain; 2 Discipline of Podiatry, College of Science, Health and Engineering, La Trobe University, Bundoora, Melbourne 3086 Australia; 3 Department of Nursing and Podiatry, University of Malaga, IBIMA, Malaga, Spain

**Keywords:** Paediatric research, Risk factors

## Abstract

The purpose of this study was to analyze spatiotemporal parameters of gait in children using varyingly loaded Backpacks(BP). This cross-sectional study examined 231 schoolchildren (118 boys, 113 girls) aged six to 12 years, carrying a traditional BP to manipulate loading (Crossing Backpack Children Arpenaz 7 Litres, Junior Red Quechua). Load was added to the BPs in increments of 5%, 10%, 15% and 20% of the child’s body weight. Spatio-temporal parameters were measured with the OptoGait system. Significant differences were observed in single support (p < 0.001), and double support (p < 0.001). No statistically significant differences were observed in step length (p = 0.959) between the five loading conditions. Similarly, no statistically significant differences were found in the contact phase (p = 0.208), although significant changes were seen between baseline, 15% of body weight (p < 0.005), and 20% of body weight (p < 0.005). The effect sizes from the ANOVA in the single support was low (0.015), and double support was moderate (0.02). Increased weight in BPs reduced both children’s balance and single support, increased double support, but did not change step length. The children increase double support with heavier loads to help their balance. The spatio-temporal changes were most evident with BP loads between 15–20% of body weight. Affective responses, including the perception of heaviness or difficulty in carrying the schoolbags need to be included in further and prospective investigations.

## Introduction

Most school aged children transport their school books and equipment on a daily basis, usually using carts or backpacks (BPs). BPs are the most common method of transporting external cargo, with 90% of students using a traditional BP^1^. Schoolbag carriage is a global activity estimated to occur in 668 million primary school children^2^. In most studies, the weight recommendations range from 10–15% of children’s body mass^3,4^. Observed postural changes in both static stance and dynamic gait, have been reported with external loads greater than 20% of a child’s body mass^5^. Larger loads have been related to back pain in children^6^. Previous studies cite that between 4.7% and 38.0% of children carry loads greater than 20% of their body weight daily^3,7^. Two-thirds of children affirm the daily use of BPs when walking an average of 14.55 km to and from school each week^8^. A child’s gait can be influenced by many intrinsic factors: limb length, joint range, muscle tone^9^, neuromuscular diseases^10^; and also by extrinsic factors: footwear, clothing or carrying loads which may change their walking pattern^11^. Measures of spatio-temporal gait parameters are used to identify walking difficulties for diagnoses, and may also determine prognosis^12^. This begins early in childhood, where the phases of infant development from sitting to walking independently, represent a important milestones in the development of motor control and coordination^13^. Schoolbag characteristics are one biomechanical factor often implicated as a cause of back pain in children and adolescents. Despite the lack of convincing evidence, schoolbags have been traditionally linked to back pain in children and adolescents^14^. Heightened awareness of this issue by the general public means that clinicians are frequently asked for advice on a preferred style of schoolbag and/or how to carry the schoolbag to reduce risk of back pain^15^. Therefore, the purpose of this study was to determine which spatio-temporal gait parameters in children show significant changes due to backpack weight, and the percentage of children’s body weight which generates theses changes, with the aim of preventing postural disorders.

## Results

This experimental intra subject analysis is based on a study population of 231 children (113 girls). The average age was 9.13(1.59) years, with average body mass index (BMI) of 20.46(4.14) kg/m^2^ (Table 1). Test of dominant side showed that 93% of children were right footed. In addition, limb length was found to increase with age, from seven years (average limb length 0.06(0.03) m, to 11 years (average limb length 0.78(0.04) m, but did not influence the results

**Table 1:** Characteristics of the sample (n = 231).

	Mean	CI 95%		SD	Minimum	Maximum
Age (years)	9.13	8.92	9.34	1.59	6	12
Weight (kg)	38.65	37	40.3	12.72	18.5	86
Height (m)	1.36	1.34	1.37	0.12	1.09	1.69
Size shoe (Eur)	22.31	21.99	22.64	2.5	18	35
Body Mass Index (kg/m^2^)	20.46	19.92	20.99	4.14	13.77	36.18

No statistically significant differences were observed in step length (F(1;230) = 0.88, p = 0.959) between the five measures. The difference is minimal between the baseline and the different BP loads, and the results range between 59.18 cm at baseline to 58.93 cm with a load of 20% body weight.

**Figure 1:**
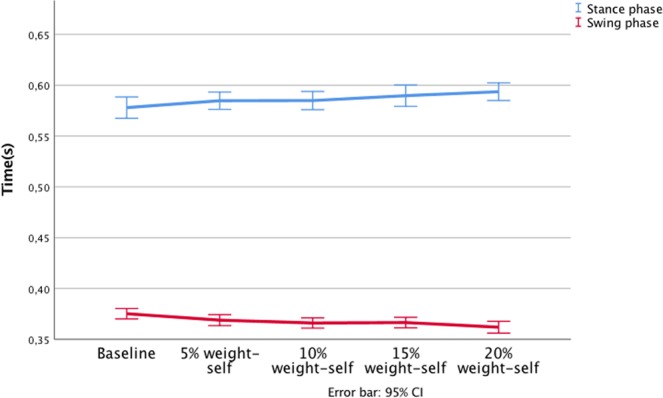
Load/time for each BP loading condition, at stance phase and swing phase.

Further, no statistically significant difference was observed between the BP load conditions contact phase time (F(1;230) = 1.47, p = 0.208). However, significant changes were observed in the measure of midstance at baseline, with BP load of 15% body weight and 20% of body weight (F(1;230) = 9.88, p < 0.005), with a 0.02 second time lag between baseline and the 20% load (Fig. 1).

**Figure 2:**
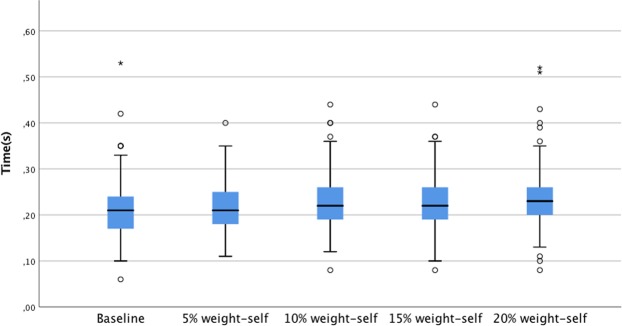
Double support phase showed linear increase with greater BP loads.

**Figure 3:**
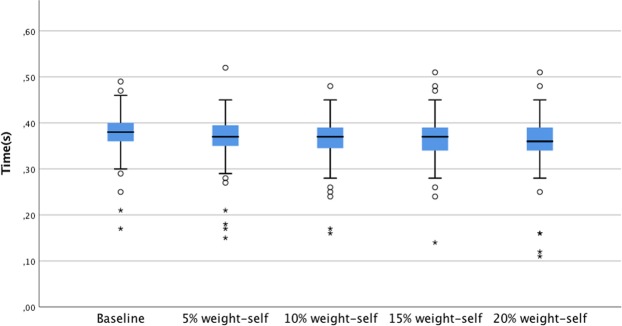
Single support phase showed reducing trend as BP loads increased.

In addition, single support showed a decrease of 0.02 seconds (F(1;230) = 8.27; p < 0,001) (Fig. 2), in contrast with double support, which showed an increase of 0.02 seconds (F(1;230) = 7.04: p < 0.001) (Fig. 3). The effect sizes from the ANOVA in the single support was low (0.015), and double support was moderate (0.02) (Table 2).

**Table 2:** Backpack weight and spatiotemporal parameters.

	Baseline	5% weight-self	Mean(SD)	15% weight-self		F(4.95)	η^2^
			10% weight-self		20% weight-self		
Step length(cm)	59.18 (6.94)	58.75 (7.20)	58.96 (7.06)	59.20 (7.18)	58.93 (7.54)	0.88	0.004
Stance phase(s)	0.58 (0.7)	0.58 (0.7)	0.58 (0.7)	0.59 (0.7)	0.59 (0.7)	1.47	0.02
Swing phase (s)	0.38 (0.4)	0.37 (0.4)	0.37 (0.4)	0.37 (0.4)	0.36 (0.4)	9.88*	0.04
Single support(s)	0.38 (0.4)	0.37 (0.4)	0.37 (0.4)	0.37 (0.4)	0.36 (0.5)	8.27**	0.015
Double support(s)	0.21 (0.5)	0.22 (0.5)	0.22 (0.5)	0.23 (0.5)	0.23 (0.6)	7.04**	0.02

*p < 0.01, **p < 0.001.

## Discussion

Our study aim was to determine the influence of weight inside of BP on the phases of the gait cycle, for children walking over ground at a self-selected velocity, taking into account the largest possible number of spatiotemporal gait parameters, and using the Optogait measurement system. Although each participant intuitively decided his/her own walking speed, as was found most comfortable, there were no significant differences in this respect.

The stance load response and swing phases all had a significantly greater duration for the 15 and 20% weight children than for those with baseline weight. The use of BPs in school children is very common, with many carrying loads for 15 minutes to and from school everyday. With more than 50% of school children found to carry loads higher than the recommended limit, this activity has public health implications^1^. Adding weight to the body using a BP, will shift the centre of gravity toward the rear of the base of support (feet). This combination of increased load and postural change can alter gait patterns. In our study, we compared the influence of BP loads with the spatiotemporal gait parameters in typical children using the Optotgait.

In this study varying loads were used, in attempt to ascertain the critical load at which spatio-temporal changes occur, as compared to baseline, with an effect size moderate. Although a linear dose-response curve was not detected, identified gait changes with BP loads of 15% body weight were evident in the study sample of school children.

Most other studies agree that external loads influence body posture^16^, and our results also indicate that children’s BP loading should not exceed 10–15% of their body weight^17,18^. Whilst average loads vary greatly between studies, the majority of reports indicate that the usual loads, carried daily by school children, are greater than the recommended limits^7,18^.

Consistent with previous studies^4,19,20^ we found increase in both double limb support and stance phase, single support, swing and contact phase, from baseline to loads of 15% and 20% body weight. Our results indicate that carrying a BP requires children to increase the time spent on both feet to manage this load during gait. It appears that increasing the base of support via increased double limb phase, results in a better distribution of load, and maintained postural stability^3^.

In previous studies, carrying a BP was found to reduce stride length and increase stride frequency^20,21^. These findings were not returned in our study, and similarly were also not found in another similar study^1^. In our study, no variations in walking speed were observed, although two specific gait strategies were appreciated. Firstly, when loaded, some children walked more slowly to manage the weight, yet secondly and by way of contrast, other children increased their speed in response to loading.

In a recent report^22^, an investigation compared spatio-temporal gait parameters between normal weight, overweight and obese children. The authors concluded no difference in speed, yet conversely, a clearly significant change in gait with increased duration of both load response and pre-swing phases, but not swing phase. These findings support the idea that external loads may generate different behavioural strategies for balance stabilization than seen with child obesity. With regard to modifying velocity in response to external load^23–25^, our hypothesis is that this may be dependant on the proprioceptive and neuromuscular reactions in every child, but further research is necessary.

The occurrence of back pain, muscle fatigue and spinal deformity due to BP use in school children has been identified, although other studies find no relationship between BP weight and back pain^26,27^ in older secondary school children. BP use increases rectus abdominis activity and decreases the activity of spinal erector muscles. One study^28^ found that weights greater than 10% of body weight produced shoulder discomfort.

Some studies have used backpacks with chest straps, and found better spatio-temporal adaptation to weight gain^3^, but as 90% of students report using the traditional BP^1^, we adopted this BP style for greater external validity.

The use of school trolleys has been found to produce fewer ankle, hip, pelvis and spine adaptations than traditional BPs, using same loads (15% body weight), with kinematic patterns more comparable to unloaded walking controls^7^.

A limitation of this study is that children usually carry BPs for periods far longer than the testing sessions, negating the effects of fatigue being identified. In addition, whilst the children used their usual school shoes, and the biomechanical parameters of footwear most commonly reported in the literature are spatio-temporal, we do not know if different shoes may modify these parameters, either advantageously or deleteriously. To date, all reports investigating the effects of BP loads in children have been undertaken in shod walking settings and therefore the influence on habitually barefoot children remains unknown^26,28^. Although changes of movement in frontal plane were not analysed, previous studies suggest an increased step width in obese children^29,30^, and with increased loads^31^, but less deviation than sagittal plane changes^23^.

A recent systematic review summarised the evidence from prospective studies^15^, and concluded that schoolbag characteristics such as weight, design and carriage method do not increase the risk of developing back pain in children and adolescents. Included in this review were two prospective studies which reported that the perception of heaviness or difficulty in carrying the schoolbag were associated with back pain and persistent symptoms^15^. Pain, perception, and suggestion are complex experiences^32^. The Hawthorne effect may be active in study participants, who are expectant and alerted to possible effects^33^.

## Conclusions

In our experimental study, increased BP loads were shown to alter gait parameters. These changes indicate that children respond to heavier BP loads by increasing the double support gait phase. Spatio-temporal changes were most evident with BP loads between 15–20% of children’s body weight. The findings of a recent systematic review, indicate that children’s perception would be a helpful inclusion in further studies. Given the regular and global use of BPs by school children, and the common practice of carrying heavier than recommended loads, this study has great relevance for wider paediatric health, and requires further clarification.

## Method

### Design

**Figure 4:**
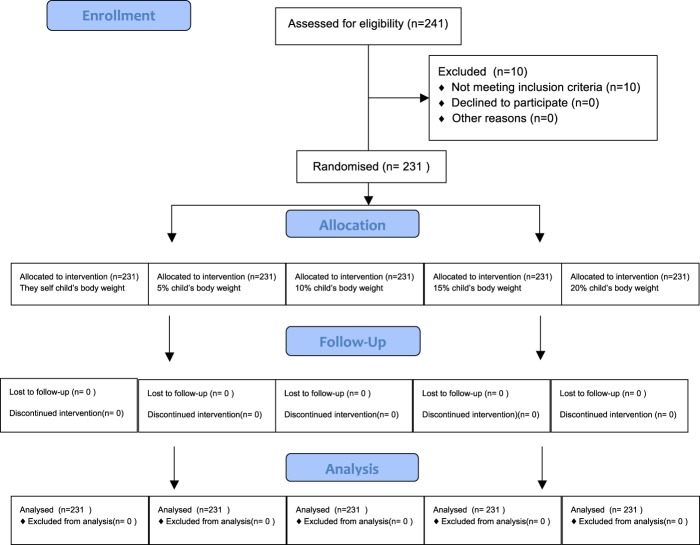
CONSORT flow diagram.

An experimental, cross-sectional design was conducted[supplementary CONSORT check list]. It is registered in the Registered at clinicaltrials.gov (NCT03839836 02/11/2019) and was carried out in accordance with the CONSORT statement (Fig. 4)[supplementary research protocol].

### Participants

We examined 231 schoolchildren (118 boys, 113 girls), aged 6–12 years, at schools in the Spanish provinces of Malaga, from December 2017 to September 2018.

The inclusion criteria were: age between 6–12 years, no pain in the lower limb and back at the time of examination. The exclusion criteria were: recent injury to the lower limb and back, alterations in the foot bones, congenital structural changes to the ankle, flatfoot associated with cerebral palsy, surgical treatment of foot or lower leg, or any genetic, neurologic or muscular conditions.

**Figure 5:**
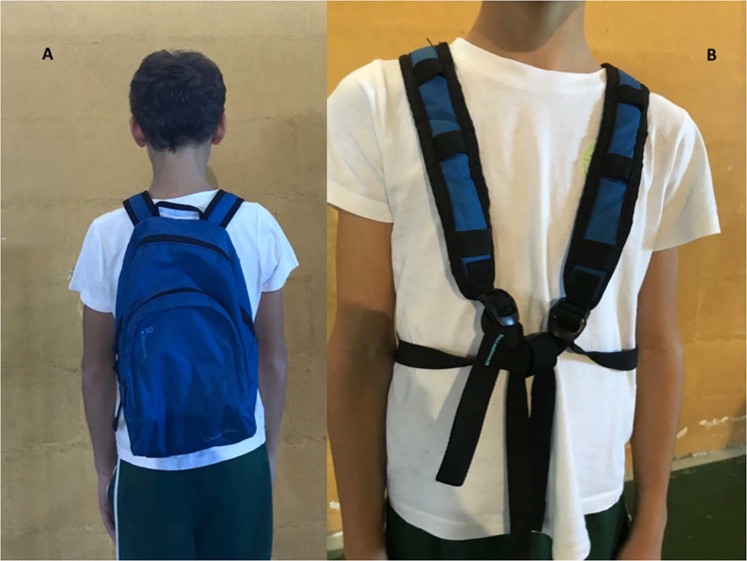
Children’s BP position shown from front (**A**) and back (**B**).

A traditional BP (Crossing Backpack Children Arpenaz, 7 Litres, Junior Red Quechua) was used to carry the specific weighted loads. Load was added to the BPs in increments of 5%, 10%, 15% and 20% of the child’s body weight, spanning those recommended in the literature^19,34^. All loads were evenly distributed in the BPs, placing the heaviest weight low and close to the spine. The shoulder straps were adjusted for each child’s height to place the BP above the hips at the low back. Sternum straps were not present (Fig. 5).

### Outcome measures

Spatio-temporal parameters were measured with the OptoGait system (OptoGait; Microgate, Bolzano, Italy). The Optogait is a reliable and validated system^35–37^, which records spatio-temporal parameters. For this study, a five-metre instrumented walkway was used, consisting of five transmission bars and five reception bars, with a separation of 120 cm. Each bar (100 cm × 8 cm) contains 96 light emitters 3 mm from the ground. Optical sensors operate at a frequency of 1000 Hz, with accuracy of 1 cm, to detect the spatio-temporal parameters related to walking, running and other movements. The software used was OptoGait v.1.11.1.0. The Optogait system was calibrated and checked for accuracy at all times, and provided an exhaustive, reliable measurement of the spatiotemporal phases of the gait cycle. Considering Initial contact (Phase1): time from initial ground contact (1 LED activated is needed to be considered) to foot flat (the number of LEDs activated stays steady ±2 LEDs). Midstance (Phase2): time from foot flat to initial take-off. During this phase, the number of LEDs stays steady ±1 LEDs. This phase finishes when the heel comes off the ground and the number of LEDs is reduced ≥2 or Propulsion (Phase3): time from initial take-off (the number of LEDs is reduced ≥2) to toe-off (when forefoot comes off the ground and the number of LEDs is 0). After data collection, data was re-filtered in the OptoGait software (Gait IN and OUT filter) with eight more filter settings (i.e., 1_1, 2_2, 3_3, 3_4, 4_4, 4_5, 5_4 and 5_5). Together with the default setting (i.e., 0_0), a total of 9 filter settings were considered in the current work.

### Procedure

Two researchers (JPM and JMA) independently interviewed the patients to obtain the study data. The demographic data was conducted in one area, where children’s height and weight were measured (weight to the nearest 0.05 kg using calibrated Digital Pegasus Scales). In another area, children were measured using the Optogait system.

Gait parameters were assessed using the protocol for the Optogait reliability testing^19^ The test-retest inter-examiner reliability of Optogait presents good correlation, ranging from 0.785 to 0.952. The coefficient of variation of method error was low, ranging from 1.66% to 4.06%, and all parameters had standard errors of measurement between 2.17 and 5.96%, indicating strong reliability.

**Figure 6:**
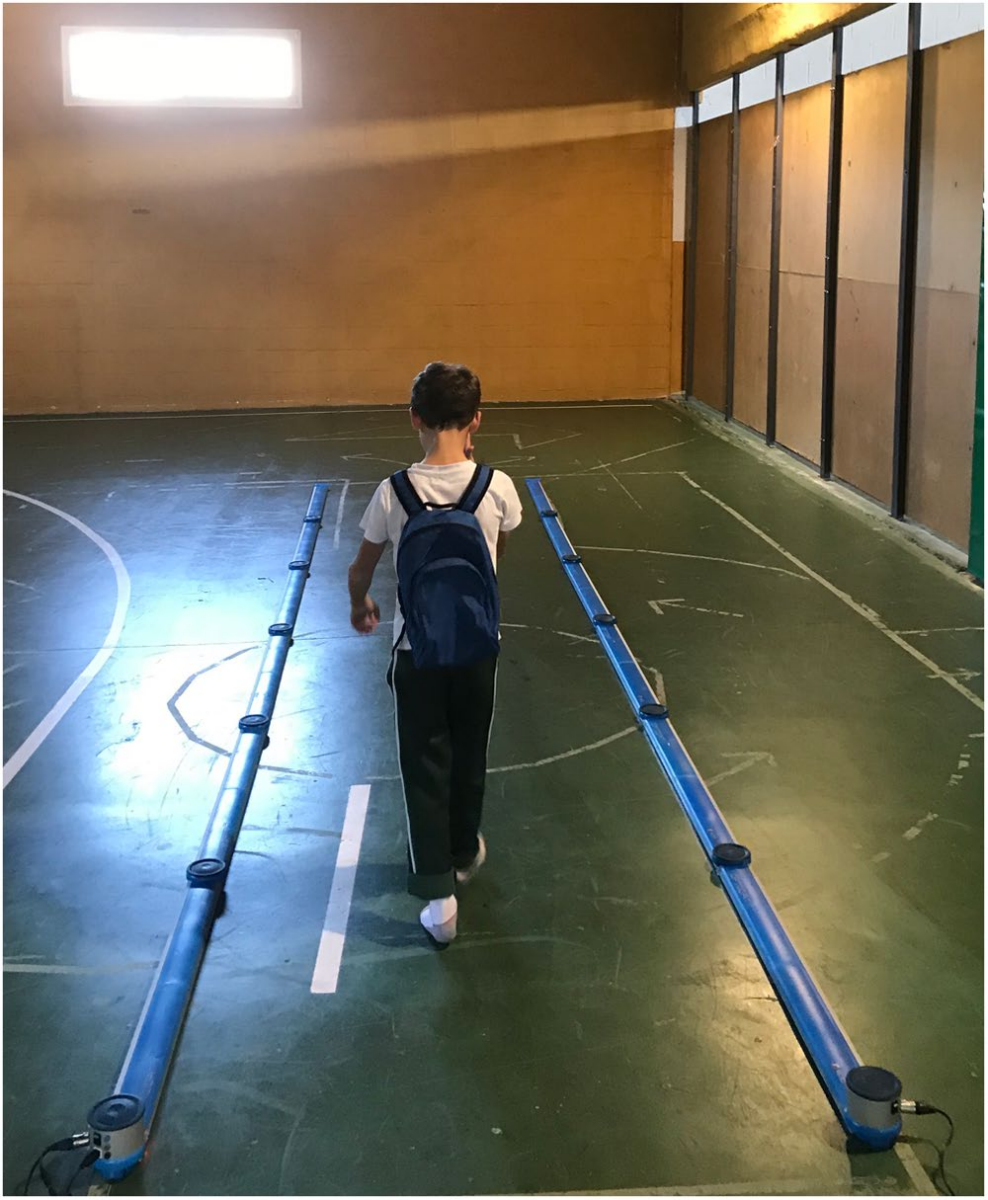
Child with loaded BP on the Optogait track.

Assessment of gait involved the children walking on a walkway at a self-selected speed, repeated three times, and calculating the mean speed. Prior to data collection, the children performed one familiarization trial for five minutes. Children were asked to walk naturally, facing forward, with their hands out of pockets; wearing light, comfortable clothes, with the BP placed correctly. As the children started walking, the researchers selected the foot of the first step inside the bars, in the Optogait software. After being instructed to ‘walk slowly at a self-selected speed’, the children walked from a point two metres in front of the bar and stopped at a point two metres behind the last bar, to minimize effects of acceleration and deceleration (Fig. 6).

After this, they were instructed to turn around and walk back to the starting point. A three-minute interval occurred between individual assessments to transmit the data, as well as to prepare for the next assessment. Only steps in the sensor areas were included in the analysis. Recording six to eight strides is reported as sufficient to obtain representative data for unimpaired adults (defined as 95% confidence intervals within 5% of error)^35^, however in this study of children, ten strides were measured. After three practice trials, the data was acquired. Subsequently, five experimental conditions were measured: without BP, BP loads of 5%, 10%, 15%, and 20% of body weight, including bags weight of 0.5 kg inside of the BP. The order of these loaded conditions was randomised by software for the five measurements to be obtained.

The data entry was performed by a single research assistant, who was blind to the nature of the study. All data entry was double-checked before statistical analysis.

### Sample size

The sample size was determined by application of the EPIDAT program, using data from a previous pilot sample to obtain mean and standard deviations for the main outcomes variables: contact phase of the heel, total contact and propulsion. Following, the study was designed to detect changes exceeding 0.8 (high effect size) with a type I error of 0.05 and a type II error of 0.2. This calculation produced a necessary sample size of 199 subjects, although in fact 231 were recruited, to cover any potential missing data.

### Data analysis

SPSS v. 25.0 program (IBM Inc., Chicago, IL, USA) was used for statistical calculations using descriptive and inferential statistical tests. The gait data was tested for normality by using the Kolmogorov–Smirnov test, set at a significance level of p < 0.05. To determine test-retest reproducibility, Intraclass Correlation Coefficients (ICC) with the 95% Confidence Interval (95%CI) was conducted in a sample of 25 children. Most parameters showed high reproducibility (>0.8). Nevertheless, the foot progression angle and the base of support were slightly less reliable (ICC’s 0.71. 0.74 respectively).

The spatio-temporal gait parameters measured were step length, stride length, single limb support time, double limb support time, swing and stance phase times. A general linear model with repeated-measures was performed to compare the differences in the parameters along the five measures. Mauchly’s test was used to evaluate sphericity. In case of non-sphericity, Greenhouse-Geisser estimation was used. Bonferroni post-hoc test was performed to determine the differences among measures with weights. The effect size test was assessed with eta-square.

### Ethical approval and informed consent

The parent and/or legal guardian were provided with information about the study, a statement attesting to informed consent was sign. The children were fully informed of the procedures involved and gave assent. All procedures were in accordance with the ethical standards of the institution and the experimental protocol was approved by a named institutional of University of Malaga (CEUMA 91/2016H) and with the 1964 Helsinki declaration.

## Supplementary information

Consort Check list

Research protocol
