# Supplementary material for: Variation of spatiotemporal parameters in school children carrying different backpack loads: a cross sectional study
Source: Sci Rep. 2019 Aug 21;9:12192. doi: 10.1038/s41598-019-48675-3 (PMC6704062; doi:10.1038/s41598-019-48675-3)
Supplement: Supplementary file 2 — Research protocol [file 41598_2019_48675_MOESM2_ESM.docx]

**Research Protocol**

**Variation of spatiotemporal parameters in school children carrying different backpack loads: a cross sectional study**

- Name and title of the investigator(s)
- Joaquin Paez-Moguer Department of Nursing and Podiatry. University of Malaga, Spain ([joaquinpaez@uma.es](mailto:joaquinpaez@uma.es))
- Jesus Montes-Alguacil Department of Nursing and Podiatry. University of Malaga, Spain ([jesmonalg@uma.es](mailto:jesmonalg@uma.es))
- Irene Garcia-Paya Department of Nursing and Podiatry. University of Malaga, Spain ([irenegpaya@uma.es](mailto:irenegpaya@uma.es))
- Miguel Medina-Alcantara Department of Nursing and Podiatry. University of Malaga, Spain ([migmedalc@uma.es](mailto:migmedalc@uma.es))
- Angela Margaret Evans Discipline of Podiatry, College of Science, Health and Engineering, La Trobe University, Bundoora, Melbourne, 3086, Australia ([angela.evans@latrobe.edu.au](mailto:angela.evans@latrobe.edu.au))
- Gabriel Gijon-Nogueron Department of Nursing and Podiatry. University of Malaga. IBIMA. Spain ([gagijon@uma.es](mailto:gagijon@uma.es))

**Rationale & background information**

## Back Packs are the most common method of transporting external cargo, with 90% of students using a traditional BP. In most studies, the weight recommendations range from 10-15% of children’s body mass ^3,4^. Observed postural changes in both static stance and dynamic gait, have been reported with external loads greater than 20% of a child’s body mass ^5^. Larger loads have been related to back pain in children^6^. Previous studies cite that between 4.7 % and 38.0% of children carry loads greater than 20% of their body weight daily ^3,7^. Two-thirds of children affirm the daily use of BPs when walking an average of 14.55 km to and from school each week ^8^.

References

**Study goals and objectives**

## The purpose of this study is to analyze the changes of the spatio-temporal parameters of gait, in school children, with five specific load conditions in a typical BP because we can detect which it is the limit weight in the BPs in the kids to go to the school without change in their gait .

**Study Design**

An experimental, cross-sectional design will be conduct. We will examine schoolchildren between aged six to 12 years, at schools in the Spanish provinces of Malaga, from November 2018 to January 2019. The inclusion criteria will be: age between six and 12 years, no pain in the lower limb and back at the time of examination. The exclusion criteria will be: recent injury to the lower limb and back, alterations in the foot bones, congenital structural changes to the ankle, flatfoot associated with cerebral palsy, surgical treatment of foot or lower leg, or any genetic, neurologic or muscular conditions.

**Methodology**

### *Procedure*

### Two examiners will be responsible for the system software and collecting data. General information, including height and weight, will be recorded. To measure gait parameters, the protocol use for the Optogait reliability testing will be used^17^. This involved the children walking on a walkway at a comfortable speed, repeating this walk three times and calculating the mean speed. Prior to data collection, the children performed one familiarization trial for five minutes. Children will be asked to walk naturally, facing forward, with their hands out of pockets; wearing light, comfortable clothes, with the BP placed correctly. As the children start walking, the researchers selected the foot of the first step inside the bars, in the Optogait software. After being instructed to ‘*walk slowly at a comfortable speed’*, the children walk from a point two metres in front of the bar and stopp at a point two metres behind the last bar, to minimize effects of acceleration and deceleration . After this, they will be instructed to turn around and walk back to the starting point.. Only steps in the sensor areas will be included in the analysis. Recording six to eight strides is reported as sufficient to obtain representative data for unimpaired adults (defined as 95% confidence intervals within 5% of error)^27^. Subsequently, five experimental conditions will be measured: without BP, BP loads of 5%, 10%, 15%, and 20% of body weight. The order of these loaded conditions was random.

**Safety Considerations**

The tests won’t have any problema for the kids

**Follow-Up**

The repeat of the test will be in the same moment with an interval of time between each weight

**Data Management and Statistical Analysis**

### SPSS v. 25.0 program (IBM Inc., USA) will used for statistical calculations using descriptive and inferential statistical tests. The gait data will be tested for normality by using the Kolmogorov–Smirnov test, set at a significance level of p < 0.05. To determine test-retest reproducibility, one-way ANOVA Intraclass Correlation Coefficients (ICC) with the 95% Confidence Interval (95%CI) will be conducted in a sample of 25 children.

### The spatio-temporal gait parameters measured will be step length, stride length, single limb support time, double limb support time, swing and stance phase times. Averaged values for continuous variables with non-normal distribution will be expressed as Mean ± Standard deviation (SD). The one-way analysis of variance (ANOVA) will be performed to compare the groups with Bonferroni post-hoc test, will be performed to determine the effect of different weights.

**Expected Outcomes of the Study**

The protocol will be carried out in accordance with the CONSORT statement

**Duration of the Project**

The timetible of this Project will be from June 2018 to February 2019

**Project Management**

GG-N, AM-N, and JPM will contribut to the conception of this study. GG-N, MM-A, and will do the statistical analysis. Data collectors will be collected in Spain JM-A and JPM. GG-N, AM-N,IGP, JP-M, JM-A and MM-A will be involved in the writing and in the review of the manuscript.

**Ethics**

### The parents will be provided with information about the study, and those consenting for their children to participate, also complete a questionnaire. The children will be fully informed of the procedures involved and gave assent. All procedures will be in accordance with the ethical standards of the institution and the experimental protocol was approved by a named institutional of University of Malaga (CEUMA 91/2016H) and with the 1964 Helsinki declaration.

**Informed consent**

This Informed Consent Form is addressed to the parents and guardians of the students of the Primary School to which they are invited to participate in the "Study of Spatio-Temporal Parameters of Walking in Children".

What is the study?

Children's gait measurements will be made using the Optogait system, which will analyze parameters such as step length, support time, cadence and speed, to determine the normality parameters and analyze the changes of the spatio-temporal parameters of gait, in school children, with five specific load conditions in a typical BP.

What tests will be done?

The weight, size, shoe number, foot type will be measured and a 30 "walk test will be performed with different weight in the Backpack.

Who performs the study?

Podiatry professors of the Faculty of Health Sciences of the University of Málaga

Participation in this research does not include the presence of possible side effects or unwanted effects, since they are not invasive tests, but purely biomechanical.

Your participation in this research is totally voluntary. You can choose to participate or not participate. You can change your mind later and stop participating even when you have accepted before.

With this research, something out of the ordinary is done in your community. We will not share the identity of those who participate in the investigation. The information we collect for this research project will be kept confidential. The information about you that will be collected during the investigation will be put out of reach and nobody but researchers will have access to see it.

I have read the information provided. I voluntarily consent to participate in this investigation as a participant and I understand that I have the right to withdraw from the investigation at any time without being affected in any way.

Name and surname of the participant: DNI:

Tutor Signature: Date
